# Influenza imprinting in childhood and the influence on vaccine response later in life 

**DOI:** 10.2807/1560-7917.ES.2019.24.48.1900720

**Published:** 2019-11-28

**Authors:** Alyson A Kelvin, Maria Zambon

**Affiliations:** 1 Department of Pediatrics, Division of Infectious Disease, Faculty of Medicine, Dalhousie University, Halifax, Canada; 2 Canadian Centre for Vaccinology, IWK Health Centre, Halifax, Canada; 3 National Infection Service, Public Health England, London, United Kingdom

**Keywords:** Influenza, Vaccine effectiveness, Imprinting, Glycosylation, Pre-existing immunity, Antibody

First impressions are important and often long-lasting. The first influenza virus infection during childhood, termed *immune imprinting,* is recognised for its influence on subsequent infections and vaccinations [1,2]. The *imprinting event* initiates a cascade of innate and adaptive immune responses leading to an immunological memory retained over a person’s lifetime. Recent studies in *Eurosurveillance* report potential implications for vaccine responses. Skowronski et al. with the Canadian Sentinel Practitioner Surveillance Network (SPSN) [3] and Kissling et al. with the Influenza Monitoring Vaccine Effectiveness (I-MOVE) in Europe [4] observed evidence of an age-related cohort effect reducing vaccine protection during the 2018/19 influenza season, which they infer occured as a result of an immunological imprinting event.

**Figure 1:**
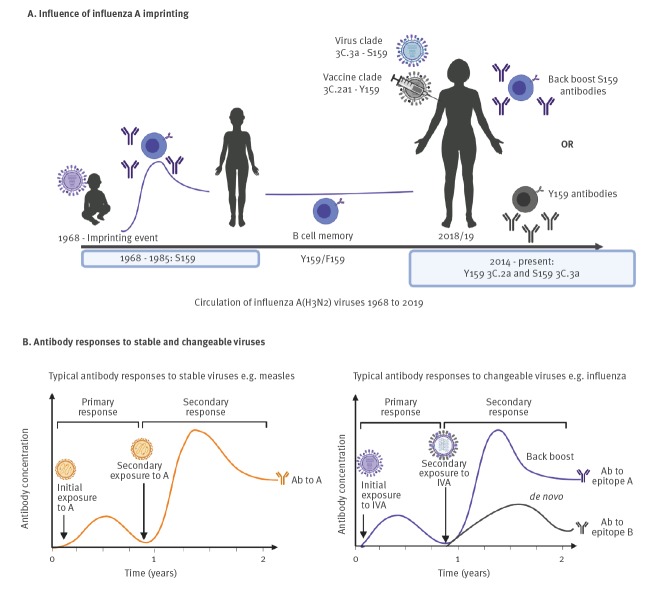
Influenza imprinting, immune refinement, and imprint-regulated effect of vaccine (I-REV)

In its publication, the Canadian group noted a relative paucity of influenza A(H3N2) cases due to clade 3C.3a among unvaccinated adults 35–54 years-old, suggesting that this cohort may have had some pre-existing protection [3]. The SPSN data also showed a paradoxically increased risk of medically attended influenza A(H3N2) illness among vaccinated adults in the same age range — a negative vaccine-associated effect that was not observed in flanking age groups or in association with co-circulating clade 3C.2a viruses. To reconcile these divergent age- and clade-specific observations, the Canadian investigators propose a unifying hypothesis they call imprint-regulated effect of vaccine (I-REV). The I-REV hypothesis is predicated on immune responses to a dominant molecular epitope shared between contemporary 3C.3a viruses in 2018/19 and influenza A(H3N2) viruses that circulated during the 20-year period following the 1968 pandemic (Figure 1A). Egg-adaptive or other antigenic changes coincidentally occurred at the same epitope in influenza A(H3N2) strains from a different clade (3c.2a) during the 2018/19 season. According to the I-REV hypothesis, immunity arising from distant childhood imprinting to the shared epitope may have protected unvaccinated adults from 3C.3a viruses in 2018/19, whereas receipt of clade 3c.2a vaccine antigen may have induced immunity that negatively interacted with the imprinted immunity [3]. In their more recent publication in this issue, the I-MOVE group similarly reports negative influenza A(H3N2) VE estimates that were pronounced for middle-aged adults and 3C.3a viruses, in keeping with the I-REV hypothesis [4].

Ab: antibody; F: Phenylalanine; IVA: influenza virus A; S: Serine; Y: Tyrosine.

Panel A: Imprinting during infancy after the 1968 pandemic with an influenza A(H3N2) virus carrying a serine (S) at position 159 in the haemagglutinin antigenic B site may have led to antibodies elicited toward this position. During the 2018/19 season, the circulating 3C.3a viruses also carried a serine at amino acid 159. A re-exposure to S159 could back-boost previously acquired antibodies from the imprint. Concomitantly, the H3N2 vaccine component from the 2018/19 season was a 3C.2a1 virus with a tyrosine (Y) at 159 which was further exposed by a loss of glycosylation at site 160 following egg adaptation. Vaccination with the egg-adapted vaccine antigen may skew immune responses and antibody generation away from targeting epitopes needed for protection.

Panel B: At the second exposure of a stable virus such as measles virus, the antibody responses are boosted toward the original antigenic sites for a faster and larger response. In comparison, circulating influenza A(H3N2) viruses are constantly changing their antigenicity through antigenic drift. The viruses retain some antigenic similarity over time but changes also occur as a result. The secondary exposure of a person who has already been exposed to an influenza virus may lead to both back-boosting of originally acquired antibodies and also the development of antibodies to new epitopes.

The concept of immunological memory recall skewing the outcome of a subsequent influenza virus infection is not new [5]. The term *original antigenic sin (OAS)* was introduced in the 1960s and has since been interpreted as the negative influence of the host’s first influenza virus infection on subsequent influenza exposure [5-7]. The *sin* in *OAS* refers to the immune system’s preference to recall pre-existing antibodies over eliciting new responses against novel antigenic epitopes [8]. The notion of exclusively negative implications of pre-existing immunity is no longer the prevailing dogma [6]. Epidemiological and seroarcheological studies demonstrate that memory responses from a first infection can also be protective, depending on the order of virus exposures [2,9-11]. Specifically, B-cell clonotypes producing cross-reactive antibodies can be detected decades later, illustrating the longevity of primary memory [9,12]. Examples include the low incidence influenza-associated medical consultations by elderly people (> 65 years) during the 2009 influenza A(H1N1) pandemic attributed to cross-reactive antibodies generated by ancestral influenza A(H1N1) viruses following the 1918 pandemic [9,13], as well as immune memory to highly conserved haemagglutinin (HA) stem regions as reported by Gostic et al. and others [2,14].

Evidence from more mechanistic studies suggests that during a secondary or tertiary exposure, the imprinted immune system can recall imprinted memory, undertake clonotype modification, and elicit de novo B-cell clonotypes reactive towards new viral epitopes [6,15,16] (Figure 1B). The continual interaction between the immune system and influenza viruses is dynamic, i.e. imprinted by the first infection but further refined at each subsequent exposure, thereby building the host immune background or preimmunity. Both Skowronski et al. and Kissling et al. report negative vaccine effects that were pronounced among participants who were repeatedly vaccinated vs repeatedly unvaccinated in 2017/18 and 2018/19. This observation may be relevant in the underlying mechanisms of vaccine interaction with preimmunity. Perhaps repeated vaccination lends opportunity for several rounds of immune refinement, shaping or narrowing of imprinted cross-protection as suggested by Skowronski et al. The phenomenon becomes understandable if it is proposed that vaccine-induced antibodies are mismatched towards key immunodominant epitopes as a result of changes at these locations arising from egg adaptation or other antigenic change in vaccine strains, which then affects the precise repertoire of induced antibodies [17]. Previous reports suggest that the immune system of the imprinted host may undergo back-boosting, epitope narrowing, and/or de novo antibody generation to reshape the immune landscape during secondary or tertiary exposures [10,18,19]. Considering these possible immune events, the molecular mechanisms of immune reshaping and potential vaccine interaction with the previously exposed immune system require experimental investigation.

Identifying serological correlates of protection against natural influenza infection or following vaccination and understanding how different vaccines can influence some of these immune responses has become imperative in the current attempts to improve vaccine effectiveness (VE), particularly in the elderly population. Recent detailed studies on immunological repertoire, using human monoclonal antibodies derived by molecular cloning from plasmablasts found in elderly adults after vaccination, indicate reduced somatic recombination events during immunoglobulin maturation, limiting diversity generation [20]. Other studies using a similar approach, which studied individuals longitudinally over several seasons of vaccination, demonstrate a bias towards expansion of pre-existing B-cell clones and a predominance of antibodies of lower potency directed towards highly conserved stem regions of HA [21], which may explain some of the observations of back-boosting, imprinting and OAS. Such detailed studies, dissecting monoclonal antibody responses of a few individuals after vaccination, provide a useful theoretical framework which can be used to interpret what is being observed in clinical practice. New influenza vaccines such as adjuvanted and mammalian cell-derived vaccines are starting to be used, with the expectation that there will be some incremental improvements in vaccine performance. A more detailed description of the pre-existing or preimmune serological profile that associates with illness and severity, especially in vaccine failures, will help understand the relationship with VE and assist with inferences about population susceptibility.

Synthesising the I-REV hypothesis with imprinting evidence from the literature, is a reminder that the foundations of our understanding of imprinting lies in the interpretation of observational data. From the recent reports by Skowronski et al. and Kissling et al. to the studies by Gostic et al. and Worobey et al. and older accounts of OAS [2-4,7,8,15], there has been frequent observational evidence of the influence of influenza history on current infections and vaccinations in humans. However, the validation of these specific observations by mechanistic experiments has not been as complete. Cross-protection studies in animal models have been plentiful, but these studies have not often appropriately recapitulated the variables of the human data, mainly because of the lack of appropriate reagents. Too many of these studies have relied on mouse-adapted influenza viruses which do not accurately represent the viral antigenic variations that cause human clinical disease [22]. Furthermore, infection–reinfection animal studies often neglect the developmental time course of immunological memory. The adaptive humoral response follows clonal expansion and contraction, leading to memory establishment over several weeks to months: Immune maturation on this time scale is rarely recreated in laboratory studies which can therefore not be representative of human seasonal influenza virus exposure and immune memory maturation [23].

Improving influenza vaccines would benefit from greater attention to human imprinting studies, leveraging animal models with more human-like influenza histories or technologies that better capture immune specificity and virus evolution in humans. Implementing these will help identify specific viral epitopes of critical importance to protective immunity or better model responses to current or novel vaccines [18,24,25]. Routine sequencing of clinical viral isolates will provide a detailed snapshot of what is circulating and capture prominent viral amino acid changes potentially influencing immunity although the ability to predict antigenic cluster transitions is very limited. The choice of candidate vaccine strains is based on recognition of emerging strains and expanding clusters associated with disease rather than knowledge of patterns of immune susceptibility in the human population. The analysis strategy used by the Canadian group associating molecular signatures of circulating and historical viruses with observed VE could be employed to determine other potential I-REV events. This requires greater granularity in VE estimation and fuller characterisation of viruses in the cases and controls contributing to VE estimates. Although the Skowronski et al. report suggests a negative interaction of the imprinted immune system in a particular age group with a specific vaccine composition, we should keep in mind, as these authors have also underscored, that this observational phenomenon should be validated in the laboratory with appropriate models and in other human studies to provide mechanistic insights which move us from observation to response. Designing future human cohort studies in upcoming influenza seasons with appropriate biological sampling to look closely at imprinting to pivotal HA epitopes (e.g. at position 159) will be an important investigational step [3,4].

Clinical trials have begun for next generation influenza vaccines, less reliant on egg-based platforms and with the promise of eliciting more broadly reactive antibody repertoires. In light of these recent reports and increasing prominence given to understanding antibody landscapes [1,2,26], we should now recognise that new vaccines require evaluation taking into account complex and diverse host influenza histories and seasonal influenza virus circulation. Elucidating the viral epitopes and immune mechanisms dictating refinement or reprogramming during continual exposures, such as the effects reported in *Eurosurveillance* this month, should be addressed. For any individual throughout life, there may be multiple influenza infections. Now with the expansion of annual immunisation programmes, there are many more vaccine antigen exposures to consider over that period. The hierarchy of immune preference or immunodominance may depend on the order of infection or exposure with a distinction between the first and subsequent infection [27]. By viewing the history of influenza virus circulation through the lens of imprinting, assumptions can be made about the virus that imprinted on a person by cross-referencing their birth year with the predominant influenza strain bearing signature epitopes circulating at that time [2,3,8,9]. This may supplement predictions about susceptibility to infection or vaccine responses and may help to develop precision vaccination strategies.

How can we make sense of complexity in the context of needing to maintain confidence in influenza vaccines? The consistency of the observed effects reported in these two primary care studies and also suggested elsewhere among inpatient and outpatient settings (e.g. the United States) lends credibility [28]. However additional study designs are necessary, involving the range of clinical end points such as medically-attended outpatient, hospitalisation or critical care admission that influenza vaccines are intended to prevent [29]. Age- and clade-specific dips in vaccine performance as described by Skowronski et al. and Kissling et al. signal opportunities for improvement and reinforce the need to develop new vaccines capable of inducing broader and more cross-reactive protective immunity. Importantly, however, clade 3C.3a infections comprised only a small proportion of cases in both studies, limiting their contribution to a reduction in overall VE [3,4]. As well, the effect was not observed at all for the other vaccine components. As authors of both studies underscore, receiving influenza vaccine remained beneficial overall at 56% and 67% for the H1N1 component in 2018/19 as in other seasons [3].

In summary, we suggest that the potential impact of distant influenza immune imprinting on current vaccination outcomes should be considered in the design of next generation or universal vaccine candidates. The I-REV hypothesis, methods and underlying mechanisms may be of future use for identifying particularly susceptible cohorts or for targeting improved immunisation strategies, and may contribute to vaccine strain selection including considering options for avoiding egg adaptation in candidate vaccine strains. While I-REV may contribute to improved scientific understanding and enhanced vaccine development in the long term, considering our current vaccine programmes and the total protective effect of vaccine, the benefits of continued seasonal influenza vaccination in recommended groups cannot be overstated.
